# Passive RFID-Based Inventory of Traffic Signs on Roads and Urban Environments

**DOI:** 10.3390/s18072385

**Published:** 2018-07-22

**Authors:** José Ramón García Oya, Rubén Martín Clemente, Eduardo Hidalgo Fort, Ramón González Carvajal, Fernando Muñoz Chavero

**Affiliations:** 1 Department of Electronics Engineering, University of Seville, 41092 Seville, Spain; ehidalgo@us.es (E.H.F.); carvajal@us.es (R.G.C.); fmunoz@us.es (F.M.C.); 2 Department of Signal Theory and Communications, University of Seville, 41092 Seville, Spain; ruben@us.es

**Keywords:** inventory, infrastructure-to-vehicle, location systems, radio propagation, RFID, traffic signs, urban environment

## Abstract

This paper presents a system with location functionalities for the inventory of traffic signs based on passive RFID technology. The proposed system simplifies the current video-based techniques, whose requirements regarding visibility are difficult to meet in some scenarios, such as dense urban areas. In addition, the system can be easily extended to consider any other street facilities, such as dumpsters or traffic lights. Furthermore, the system can perform the inventory process at night and at a vehicle’s usual speed, thus avoiding interfering with the normal traffic flow of the road. Moreover, the proposed system exploits the benefits of the passive RFID technologies over active RFID, which are typically employed on inventory and vehicular routing applications. Since the performance of passive RFID is not obvious for the required distance ranges on these in-motion scenarios, this paper, as its main contribution, addresses the problem in two different ways, on the one hand theoretically, presenting a radio wave propagation model at theoretical and simulation level for these scenarios; and on the other hand experimentally, comparing passive and active RFID alternatives regarding costs, power consumption, distance ranges, collision problems, and ease of reconfiguration. Finally, the performance of the proposed on-board system is experimentally validated, testing its capabilities for inventory purposes.

## 1. Introduction

Road maintenance companies keep a record of the location and date of installation of all the traffic signs placed on the roadways, which helps to determine which of them have to be replaced for age reasons, as well as to plan the installation of new traffic sign infrastructure. These records soon become out of date because a large number of traffic signs disappear annually due to accidents or acts of vandalism. To keep the inventory system updated, traffic routes are regularly inspected by vehicles equipped with video cameras. Traditionally, the detection of the traffic signs in the images has been performed offline by human operators, which is expensive and time-consuming. Computer-based traffic sign detection and recognition seems to be an interesting alternative that has been widely used for applications such as speed control [1] or detection of road intersections [2] and lanes [3]. However, its performance can be degraded when the signs are partially hidden, as often occurs with urban facilities [4], or if the quality of the images is poor, which happens, for example, when the weather is adverse or in conditions of darkness [5]. Therefore, recent research has been proposed to overcome these limitations, by proposing novel approaches, such as those based on 3D recognition and location of traffic signs [6], redundant multiple-view architectures combining 2D and 3D techniques [7], or by collecting information from databases such as Google Street View with location purposes [8]. These approaches have also contributed to automatize all the inventory process and reduce the need for manual assessments of the captured images, thus reducing the post-processing times and costs, as well as the sensitivity to human errors, increasing the number of inspections that can be performed. However, even though accurate detection has been reported (94.63% in [8]), the computational burden of image-processing approaches still remains as a limiting factor, especially when implementing the most novel and accurate techniques, such as 3D reconstruction algorithms [6]. Furthermore, note that the inspection vehicle usually travels at a low speed, around 60 Km/h on roadways, because it also has to measure the camber of the road and other parameters. Therefore, to prevent affecting the traffic flow, it would be desirable to conduct the inspections at night; however, this is hardly compatible with capturing accurate images, which are detected at day in the previously referenced works [6,7,8].

The alternative to video-based inventory systems may be based on radio-frequency identification (RFID) technology. Basically, it requires placing a RFID tag at each traffic sign or urban facility, and a RFID reader in the inspection vehicle, whose purpose is to scan for RFID tags in its coverage area. RFID is a mature technology that is successfully used in many industries to track and manage goods and merchandise, while avoiding the inherent disadvantages of video-based systems. More specifically, the last decade has witnessed a flurry of research for applying RFID technology to the development of smart roads, incorporating RFID to traffic management applications. For example, the use of RFID for electronic toll collection at highways, for access control systems of vehicles in parking lots, to control traffic congestion, or to restrict traffic in designated areas in cities are well-known. A summary of different RFID based recent approaches for road applications is listed in Table 1.

In particular, RFID technology has been proposed to traffic sign detection in the last decade [9,10,11,12,24], as part of the new-generation driving-aid systems. Specifically, [9] presents an advanced driver-assistance system (ADAS) in which in-pavement passive RFID tags transmit information about the road conditions (speed limits, curve warnings, …) to the passing vehicles, which can automatically adapt their speed in response. However, since the tags are placed on the pavement, not on the traffic signs, this system is not appropriate for the intended inventory purposes. The experimental assessment of [9] confirms that the tags can be only detected at a short distance, achieving a maximum communication range of 40 cm, and the system limits the speed of the cars to 20 Km/h. An improved version of [9], which reduces the number of RFID tags required to work by a more elaborated encoding mechanism, has been presented in [24]. Furthermore, active RFID technology has been proposed in [10,11,12] to increase the transmission range on ADAS systems. Contributions [11,12] propose the attachment of active RFID tags at the traffic signs as part of high-accuracy vehicle speed-controller systems, which warn the driver of the proximity of speed limit traffic signs and at intersections and other critical points in the city. Specifically in [12], active tags are used to ensure their detection from a distance large enough to permit the operation of a car’s speed fuzzy logic controller. The system shows a good performance at low driving speeds (less than 24 Km/h). An additional feature of [12] is the use of two independent RFID readers, which reduce the occurrence of occasional missed tag detections in comparison to the case of employing only one reader. A more exhaustive comparison with [9,10,11,12,24] will be presented in Section 5, from the obtained experimental results. Active RFID has also been tested in vehicular routing applications as a substitute for GPS when the reception of GPS signals is not possible [13,14]. In these approaches, RFID tags are arranged in the road or on both sides of a tunnel’s edges, and vehicle location techniques based on the received RFID signal strengths are presented. Specifically, GPS errors are more common in dense urban areas, requiring complex positioning algorithms to achieve an effective position triangulation [25]. Finally, the growing interest in addressing the challenges of the internet-of-things (IoT) [15,26] and infrastructure-to-vehicle (I2V) communication systems [27,28] brings us to the conclusion that embedding RFID technology in road facilities is likely to become a reality in the near future [28].

Based on the experimental data reported in previous works, such as [10,11,12,13,14], we can be confident in that active RFID technology ensures a high coverage range even if the reader is placed in a vehicle that runs at high speed, thus fulfilling the potential requirements for a traffic sign inventory system. Nevertheless, the advantages of using a RFID-based architecture would be even more pronounced if passive, not active, RFID were used, as maintenance costs would be greatly reduced in that case (passive RFID does not require battery replacement) and the price per passive tag is insignificant compared to the traffic sign’s price. A more exhaustive comparison regarding costs will be detailed in Section 2.2.3.

Although there are previous works based on passive RFID for road applications [15,16,18,19,20,21,22,23], they are mainly focused on vehicle location [15,19,22] or vehicle identification [20,21,23] purposes. Vehicle location systems require placing multiple tags on the floor to accurately locate the vehicle passing over them, and vehicle identification systems require placing the reader just on the floor to identify the tag attached to the vehicle passing over the antenna. Thus, the required distance range for both applications is lower than that required for a traffic sign inventory application. Some features of these approaches will be discussed in Section 5. Additionally, other passive applications working at different frequency bands, such as [16,18], present low distance ranges (<10 cm) as well.

Therefore, some doubts may arise about the adequacy of passive RFID for the intended tasks. Its lower coverage range, which leads to short tag detection times, more noticeable when the vehicle’s speed increases, may prevent the traffic signs from being identified during the normal operation of the system—even considering the recent improvements on the aforementioned limited read range of passive RFID tags [29] or on their performance on metallic environments [30,31]. As this problem has not been studied in the previous RFID-based approaches for inventory and vehicular routing applications, the aim of the present paper is to fill this gap and validate the use of passive technologies for the inventory purposes claimed. To this end, a theoretical model of the wireless communication link for in-motion scenarios is presented in this paper, and validated at both simulation and experimental levels. A preliminary version of this research was conducted in [32], where some of the functionalities and performance of the proposed system were presented. Besides the theoretical model and its validation mentioned above, this paper presents additional contributions with respect to [32], such as an experimental comparison between passive and active RFID, and the addition of location capabilities, by taking advantage of the information that can be codified in the tags. The integration of both capabilities leads to an additional improvement over several previously referenced approaches, which only provide a solution for inventory [9,10,24] or location [13,14] applications.

The paper is organized as follows: Section 2 presents a description of the proposed system and details its implementation aspects, such as RFID tag requirements, codification of their identifiers, employed software, and cost considerations. Section 3 presents a model of the wireless RFID link, which is used to study the reliability of RFID passive technologies for this application. In Section 4, the proposed system is validated at simulation level by using the model developed in the previous section, and at experimental level by describing a comparison between passive and active technologies, and validating the final on-board integrated system. In Section 5, the results are discussed from a comparison with other related works. Finally, Section 6 brings the paper to a conclusion.

## 2. System Description and Implementation Aspects

### 2.1. System Description

**Figure 1:**
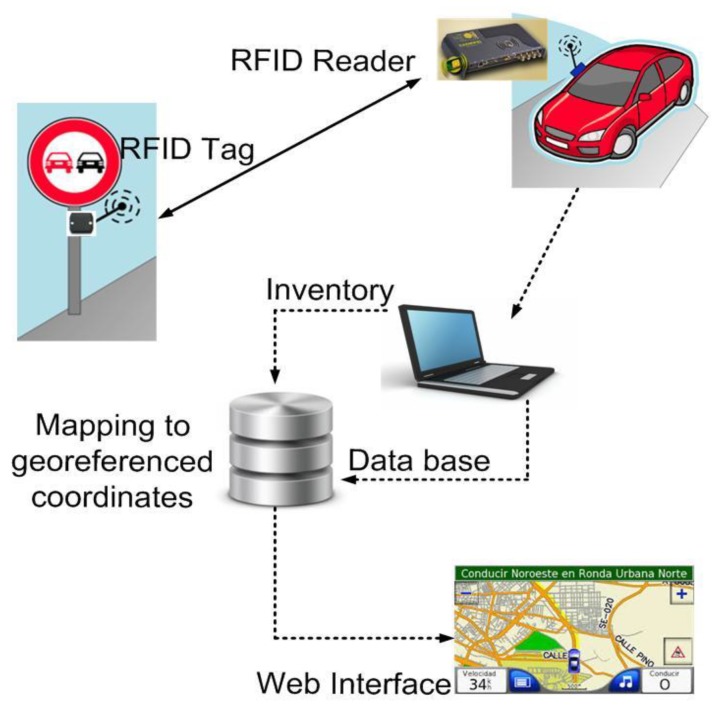
System overview.

As mentioned before, the proposed system is based on passive RFID tags on each traffic sign and an RFID reader on the inspection vehicle. Therefore, the system requires the placement, in each traffic sign, of a little inexpensive electronic tag. This tag consists of an antenna and a microchip programmed to transmit an identification code, unique to each sign, when requested to do so. The operation of the proposed system has been summarized in Figure 1: the process starts when a reader device, installed in the traveling inspection vehicle, broadcasts requests for the tags’ identifications by radio. In response, nearby traffic signs identify themselves, which enables their automatic and real-time detection. Finally, the process finishes by checking the detected tags against the original inventory in real-time, and discrepancies (e.g., the unexpected presence or absence of a traffic sign near the vehicle’s location) are reported to the road maintenance company. Additionally, the RFID identifier is mapped to geo-referenced coordinates to display the exact location of the traffic signs on a map. In principle, RFID technology makes it possible to determine the presence of traffic signs behind obstacles and in all weather conditions, even at night, which enables the physical inventory to be taken at any time. In addition, it is possible to simultaneously detect and differentiate between different tags even if they are located close to each other.

Other interesting features are that: (1) tags uniquely identify each element and can therefore provide other relevant information to the reader (e.g., the location or the date of installation of the sign); (2) no infrastructure maintenance is required; and (3) the system is highly scalable: it can be applied not only to standardized traffic signs but also to any street facilities, with inventory and location purposes.

Additionally, commercial readers are usually equipped with several antenna ports so that a set of multiple connected antennas can be used to cover multiple directions. This would enable, for example, detecting tags placed on traffic signs installed on the opposite side of the road or at the back of the signs left behind by the vehicle.

As an example of the potential offered by the system, detected traffic signs can be dynamically located on a map, so as to enable road maintenance companies to easily visualize all the traffic infrastructure. In the prototype that we have developed, electronic product code (EPC) identifiers received by the RFID reader are sent to an external computer, time-stamped, and stored on a database. Then, the traffic sign is unequivocally associated to a pair of georeferenced coordinates using the EPC frame data (whose codification is detailed in Section 2.2.1). These coordinates are used as input to display the locations of the traffic signs in a dynamically-generated map, which includes selectors and menus, providing tooltips to the map points, which give information about the inventory and location. Detected traffic signs are compared with the identifiers in the database so that all non-previously inventoried, missing, or displaced signs are easily identified. As a consequence, the information on the map can be updated automatically and in real time. The interface includes message boxes that inform about any incidences, such as a missed traffic sign, in the inventory process. These functionalities could be extended to any urban facility as well.

**Figure 2:**
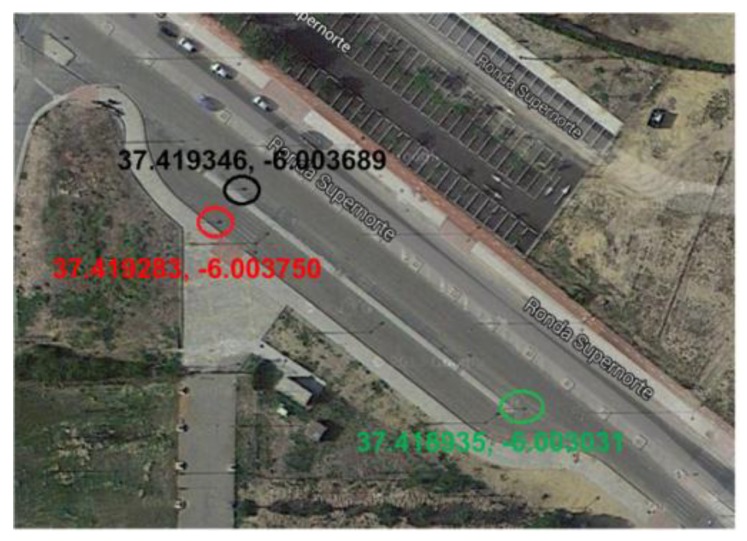
Spatial location of the detected signs.

**Figure 3:**
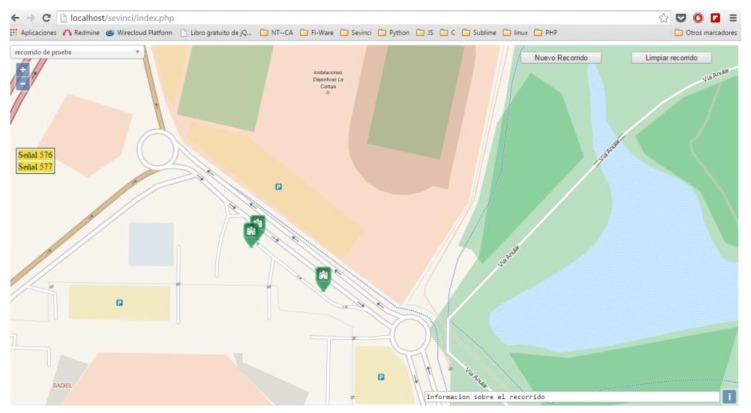
Map generated from the detected signs information.

An example of the prototype digital map is presented in Figure 2 and Figure 3. This map provides, at the same time, a visual support for the inventory functionalities and a low-cost, robust, and open-source alternative navigation system, which may be useful in various harsh scenarios for GPS communication, such as tunnels, underground, or very dense urban areas.

### 2.2. Implementation Aspects

#### 2.2.1. Tag Requirements and Data Codification

Firstly, tags must fulfill several requirements, in particular, low weight and size, suitable performance on metallic surfaces, a ruggedized package, and sufficient storage capacity for encoding different fields—such as the type of sign, road identifier, and kilometric location. These features will be detailed in Section 4.1.1, which describes the selected commercial equipment.

The implemented codification for a 96-bit EPC is described in Table 2. This encoding takes as reference the nomenclature of the Spanish roads, which are divided in stretches of 5 Km. By using this division, it is possible to implement an organized and easily upgradeable inventory.

Note that 6 bits of the 96-bit EPC are left unused, so the proposed codification would enable future extensions, or even migrations to other-countries road networks. Moreover, though this codification is oriented to road inventory applications, a similar frame could be used, previous reconfiguration of the EPC, for example, to control the inventory and the location of street facilities which can be displaced, such as dumpsters, thus opening up new opportunities to develop the paradigm of smart cities [33,34]. These capabilities for updating, extensions and migrations purposes will be ease by using passive RFID technologies which employ open communication standards and, usually, longer EPC identifiers than the active RFID case, such as described in Section 4.1.1.

#### 2.2.2. Software Architecture

The received EPC identifiers are stored on a *MySQL* database. By using the above described fields *St*, *Rd*, and *Km*, each EPC is unequivocally associated to a pair of georeferenced coordinates, which is employed as input of the dynamically-generated map and the web interface designed by using *PHP*, *Javascript*, and *JQuery*. The map view has been implemented by using the framework *Openlayers*, which enable access to the *OpenStreetMap* dataset. Additionally, the complement *Chosen* of *JQuery* has been used to design the selectors and menus.

#### 2.2.3. Costs Considerations

Costs considerations lead us to propose a system based on passive RFID technology, as described in Section 1. In passive RFID, tags collect the energy required for operation from the radio-frequency signal received from the reader device. By contrast, active RFID tags have a local power source (e.g., a battery) so that they can transmit data farther than passive tags. However, the cost per active RFID tag (around US$20–25 in the time of writing) is also much higher than that in the passive case (about US$3 for a ruggedized passive tag designed to work over metallic surfaces) [35,36]. Moreover, considering a price of around US$100–350 [37] per traffic sign, the total cost should be considerably increased installing active tags in comparison with using passive technologies. This difference regarding costs is extremely important, because the final objective of this research is to implement an inventory system for all the traffic signs which compose the Spanish National Roads Network. Therefore, placing a passive tag at each traffic sign, instead of an active tag, would be saving several million US$.

Besides the costs per tag, active technology requires batteries, which must to be replaced periodically, leading to an additional increasing maintenance costs. Additionally, active systems commonly employ proprietary communication protocols, which imply a manufacturer dependency with respect to future improvements of the system. At this point, these factors clearly tip the balance in favor of passive tags. Therefore, the following sections will describe the validation of the selected technology at theoretical, simulation, and experimental levels.

## 3. RFID Link Budget Calculation

The operation of the proposed system involves passive RFID tag readings at a maximum distance and at high speed of the vehicle inspection. To theoretically validate this idea, in this section we present a reliable radio wave propagation model that can be used to estimate the maximum range at which the tags can be detected and determine the speed limit for the inspection vehicle. The fundamentals of the RFID link budget calculation can be found in [38] for static applications. In addition, simple models have been used in the related papers [12,13] to calculate the RFID coverage range in roads. Specifically, [13] proposes an unobstructed free-space model for estimating the signal strength received from the RFID tags. A slightly more sophisticated approach is used in [12], which takes into consideration the possible interference caused by the reflection on the ground of the radiowaves. This approach serves to conclude that both the reader and the RFID tags should be as high above the ground as possible. Although not explicitly stated, a basic assumption in the propagation models in [12,13] is again that both the reader and the RFID tags are static, without movement. The contribution of this section is to generalize previous approaches in [12,13,38] by taking into account vehicle’s location and speed in the equations governing the link budget. In addition, the effect of multipath interferences in complex scenarios, such as urban environments, is modeled statistically for a more accurate description of reality. Therefore, the exposition is organized as follows: a basic background on the RFID link budget calculations (for the case of the downlink communication) is given in Section 3.1.1. The extension to in-motion scenarios under real conditions is described in Section 3.1.2.

### 3.1. Downlink (Reader to Tag) Communication

#### 3.1.1. Background

In general, the radiation power flux density averaged over time, or the average Poynting vector, of an electromagnetic wave is given by *ϕ = e^2^_rms_/120π* (W/m^2^), where *e_rms_* is the root mean square (rms) value of the electric field [39]. The effective aperture of a receiving antenna—i.e., the area over which the antenna gathers the energy of the incoming electromagnetic waves—is shown in specialized texts [39] to be *a_eff_ = g_R_λ^2^/4π* (m^2^), where *g_R_* is the gain of the antenna and *λ* (m) is the wavelength of the wave. It holds that the mean power *p_R_* delivered to the receiving antenna is the product of the Poynting vector at the antenna by its effective area *a_eff_*.

(1) $$p_{R}=\varphi \cdot a_{eff}=\frac{g_{R}\lambda^{2}}{4\pi }\frac{e_{rms}^{2}}{120\pi }[W].$$

For a free-space propagation scenario, the Poynting vector at a distance *r* (m) from a transmitter can be also calculated, for a transmission power *p_T_* (W), as *ϕ = p_T_g_T_/(4πr^2^)*, where g_T_ is the gain of the transmitter antenna in the direction of the receiver. By equating both expressions for *ϕ*, we obtain that [39]

(2) $$e_{rms}=\frac{\sqrt{30p_{T}g_{T}}}{r}[V/m].$$

Assuming, as a convenient simplification, that the dependence with time of the electric field is given by a sine or cosine function with peak amplitude √2*e_rms_*, the instantaneous field at this point is given by the wave

(3) $$e(t)=\frac{\sqrt{60p_{T}g_{T}}}{r}\cos(\omega_{c}t\prime )[V/m],$$

where *ω_c_* (rad/s) is the carrier’s angular frequency. Equation (3) reflects the fact that, as the propagation of the radio-waves is not instantaneous, the field received at time *t* was emitted at a different and earlier instant *t′*. Noting that *t-t′* is the time took by the field to propagate over the distance *r*, we can determine *t′* by the equation

(4) $$t\prime +\frac{r(t\prime )}{c}=t,$$

where *c* is the speed of light. Substituting in (3) we get

(5) $$e(t)=\frac{\sqrt{60p_{T}g_{T}}}{r}\cos(\omega_{c}t-k_{c}r)[V/m],$$

where *k_c_* = *ω_c_*/*c* = *2πf_c_*/c = *2π/λ* (rad/m) is called the ‘wavenumber’, and *f_c_* (Hz) is the frequency of the wave. 

#### 3.1.2. Propagation under Real Conditions

**Figure 4:**
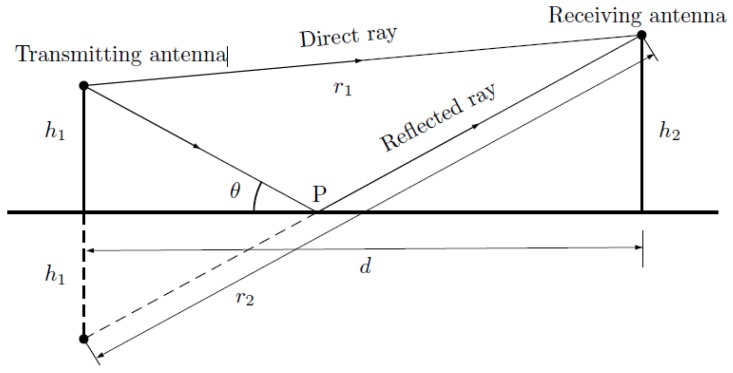
Propagation paths.

It is reasonable to suppose that the field radiated from the vehicle mainly arrives at the RFID tag via two distinct paths: one is the direct path from the vehicle to the tag, with length r_1_, and the other, of length *r*_2_, is the path reflected from the surface of the ground (see Figure 4).

Invoking (5), we model the direct field at the RFID tag as

(6) $$e_{1}(t)=\frac{\sqrt{60p_{T}g_{T}}}{r_{1}(t_{1})}\cos(\omega_{c}t-k_{c}r_{1}(t_{1})),\;$$

where *t_1_* is the time instant at which the wave received by the tag was emitted by the reader. Considering the vehicle as a point traveling at constant velocity *v* along a straight line, and after some tedious algebra, (4) yields

(7) $$t_{1}=t-\sqrt{{\left(vt\right)}^{2}+(1-{\left(v/c\right)}^{2})(d^{2}+{\left(h_{1}-h_{2}\right)}^{2})}/c,$$

where *d* is the distance from the traffic sign to the path followed by the car.

In the case of the ground-reflected path, a change in the amplitude and phase of the radiowave occurs on the reflection, which is given by the Fresnel coefficient Γ(θ) = ρ(θ)e^jΨ^ (θ), where ρ stands for the amplitude attenuation and Ψ is the phase shift. Furthermore, observe that the Fresnel coefficient not only depends on the grazing angle θ but also on the field polarization. The expression of the field produced by the reflected wave is then

(8) $$e_{2}(t)=\frac{\sqrt{60p_{T}g_{T}}}{r_{2}(t_{2})}\rho \cos(\omega_{c}t-k_{c}r_{2}(t_{2})+\Psi ),$$

where *t*_2_ is the time instant at which the ground-reflected wave was emitted by the vehicle and can be calculated similarly to that in (7).

In complex environments, such as urban areas, other reflected waves *e_3_(t)*, …, *e_N_(t)* may also reach the RFID tag. Let the *n*th wave be of amplitude *α_n_(t)* and phase *β_n_(t)*, i.e.,

$$e_{n}(t)=\alpha_{n}(t)\cos(\omega_{c}t+\beta_{n}(t)),\;$$

for *n = 3*, …, *N*. All the waves add at the receiver, leading to either constructive or destructive interference, and the resultant field is given by

(9) $$e(t)=\sum_{n=1}^{N}e_{n}(t).$$

Some algebra shows that

(10) $$e(t)=r(t)\cos(\omega_{c}t+\theta (t)),$$

where

$$\begin{array}{l} r(t)=\sqrt{i{\left(t\right)}^{2}+q{\left(t\right)}^{2}}\\ \theta (t)=\arctan(q(t)/i(t)) \end{array}$$

with

(11a) $$i=a\cdot \cos(\arctan(a_{2}/a_{1}))+\sum_{n=3}^{N}\alpha_{n}\cos(\beta_{n})$$

(11b) $$q=a\cdot \sin(\arctan(a_{2}/a_{1}))+\sum_{n=3}^{N}\alpha_{n}\sin(\beta_{n})$$

and

(12a) $$a=\sqrt{60p_{T}g_{T}}\cdot \sqrt{\frac{1}{r_{1}^{2}}+\frac{\rho^{2}}{r_{2}^{2}}+2\frac{\rho }{r_{1}r_{2}}\cos(k_{c}(r_{2}-r_{1})-\Psi )}$$

(12b) $$a_{1}=\frac{\cos(k_{c}r_{1})}{r_{1}}+\frac{\rho \cos(\Psi -k_{c}r_{2})}{r_{2}}$$

(12c) $$a_{2}=-\frac{\sin(k_{c}r_{1})}{r_{1}}+\frac{\rho \sin(\Psi -k_{c}r_{2})}{r_{2}},$$

where, for simplicity, we have dropped the dependence with time. The rms value of the field strength is given by *e_rms_ = r_rms_/√2*, where *r_rms_* is the rms value of *r(t)*. Then the mean received power can be calculated by (1), i.e.,

$$p_{R}=\frac{g_{R}\lambda^{2}}{4\pi }\frac{e_{rms}^{2}}{120\pi }=\frac{g_{R}\lambda^{2}}{4\pi }\frac{r_{rms}^{2}}{240\pi }[W].$$

To compute *p_R_*, the coefficients α and β in (11) may be also determined using ray-tracing models; however, sufficient data is not expected to be available in practice so we must characterize the data statistically. If N→∞, the central limit theorem enables us to assume that the sums $\sum_{n=1}^{N}\alpha_{n}\cos(\beta_{n})$ and $\sum_{n=1}^{N}\alpha_{n}\sin(\beta_{n})$ are zero-mean Gaussian variables with common variance *σ*^2^. Next, note that, since the wavenumber k_c_ is usually small (e.g., k_c_ ≈ 19 at f_c_ = 866 MHz), the variation of r(t) is slow in comparison with that of cos(ω_c_t) and, henceforth, in this paper we will assume that r(t) is constant, i.e., r(t) = r, at least over the time required to complete a transaction between the vehicle and the RFID tag (which is typically on the order of a few milliseconds). Under this assumption, it follows from (11) that *r^2^_rms_* = *r^2^* is the sum of the squares of two independent Gaussian variables I and Q, with means (see (11))

$$\mu_{I}=a\cdot \cos(\arctan(a_{2}/a_{1}))$$

and

$$\mu_{Q}=a\cdot \sin(\arctan(a_{2}/a_{1})),$$

where *a*, *a_1_* and *a_2_* were defined in (12). Consequently, the distribution of *r^2^_rms_* is non-centrally chi squared with two degrees of freedom, i.e.,

$$P(r_{rms}^{2})=\frac{1}{2\sigma^{2}}e^{-(r_{rms}^{2}+a^{2})/2\sigma^{2}}I_{0}(a\cdot r_{rms}/\sigma^{2}),$$

where *I_0_* is the Bessel function of the first kind and zero order. Finally, using (13), the probability that the power at the RFID tag will exceed a predetermined detection threshold *γ*, i.e., *P(p_R_ ≥ γ) = P(r^2^_rms_ ≥ γ′)*, where *γ′ = 960π^2^γ/g_R_λ^2^*, becomes

(13) $$P(r_{rms}^{2}\geq \gamma \prime )=\int_{\gamma \prime }^{\infty }\frac{1}{2\sigma^{2}}e^{-(x+a^{2})/2\sigma^{2}}I_{0}(a\cdot \sqrt{x}/\sigma^{2})dx=Q\left(\frac{a}{\sigma },\frac{\sqrt{\gamma \prime }}{\sigma }\right),$$

where *Q(·)* is the Marcum *Q* function [40]. Observe that Equation (14) gives the probability that the link will be available through the whole time interval. 

### 3.2. Uplink (Tag to Reader) Communication

If the power received by the RFID tag is higher than the predefined threshold, the tag absorbs and re-emits a fraction η of this power. As defined in the ISO/IEC 18000 family of international standards, the tag will usually employ FM0 or Miller baseband encoding techniques, as well as ASK or PSK modulation schemes, to transmit the information. The procedure to calculate the power received by the reader in the vehicle is essentially the same described in the previous section.

The presented model has been used to evaluate the reliability of the radio-link between the vehicle’s reader and the RFID tag and it will be validated at simulation level in Section 4.2 and experimentally in Section 4.3, by adjusting the design parameters to the real application.

## 4. System Validation

### 4.1. Experimental Comparison between Passive and Active Technologies

This section describes several preliminary experimental tests with the objective to present a comparison between passive and active technologies, considering the latter as the gold standard or the technology that produces the bests results (though at a much higher cost). Therefore, once we have proposed the use of passive RFID, the objective of this Section 4.1 is to validate this choice by performing similar tests to both technologies, with the objective to corroborate that, although active RFID should provide better performance (regarding coverage range or anti-collision response), using passive RFID is still possible to meet the requirements for this application, exploiting the benefits inherent to this technology. Thus, this section is organized as follows: Section 4.1.1 describes the selected equipment for both technologies, Section 4.1.2 details the performed experiments and, finally, Section 4.1.3 provides some preliminary conclusions regarding the convenience of using passive RFID, which will be corroborated later for in-motion scenarios at simulation (Section 4.2) and experimental (Section 4.3) levels.

#### 4.1.1. Equipment

##### (a) Passive components

The passive tag selected for the experiments is the Class 1 Gen 2 UHF of Confidex Ironside, which employs EPC Global 1G2 ISO 18000-6C as air-interface protocol. This tag was selected due to some features such as a low weight (22 g), enough read range on metal surfaces (up to 9 m), ruggedized package (for outdoor applications), and IP68 classification. Moreover, the tag dimensions (51.5 × 47.5 × 10 mm^3^) seem convenient for its applicability in metal surfaces, due to larger tags usually presenting a better performance in near metal environments [41], without being large enough to interfere in the visualization of the traffic sign. In addition, a critical feature of this tag is its EPC memory capacity (96–240 bits), which is necessary in order to employ this identifier for inventory and location purposes as explained in Section 2.2.1.

The selected RFID reader is the R4300P ION of CAEN RFID. Its frequency range is 902–928 MHz (FCC part 15) and 865.5–867.6 MHz (ETSI EN 302–208), using the same air-interface protocol (EPC Global 1G2 ISO 18000-6C) and a RF power up to 32 dBm (1.6 W). Besides, we use a linearly polarized antenna connected to the RFID reader, with a gain of 8 dBi, a frequency range of 860–970 MHz, VSWR (voltage standing wave ratio) < 1.5:1, and a beamwidth of 65–69°.

##### (b) Active components

The selected active tags are the Hussar Slim (HKRAT-NT02) and Garrison Rugged (HKRAT-RT02 and HKRAT-ZT02) models. Both of them use a replaceable lithium battery and transmits at 2.4 GHz. RF output is 0 dBm, presenting low power consumption (16–18 µA, 3 V). These active tags differ mainly in their application; HKRAT-NT02 is oriented to tracking and inventory applications, whereas Garrison tags find applications in harsh environments, such as metal and other non-RF-friendly items. Therefore, Garrison tags have a higher ruggedization level (meeting the IP65 standard), although they still provide a compact size (78.8 × 33.6 × 10 mm^2^). Additionally, the rugged tags have a longer lifetime (around three years without replacing the battery), so these tags could mean a reliable solution for this application, in the case of opting for active technologies.

The RFID reader is the model EMWF, from the manufacturer Empress, working at the 2.4 GHz band with a proprietary communication standard, as usual for RFID active equipment. This reader has a long range, about 30–50 m outdoors, a RF output power of 0 dBm, and a sensitivity of −85 dBm with an installed omnidirectional antenna without gain. Employing a GFSK modulation, this reader is able to identify more than 100 tags/readings simultaneously without collision, and transmit the collected data through the standards such as 802.11 b/g or TCP/IP.

#### 4.1.2. Test Setup and Experimental Results

**Figure 5:**
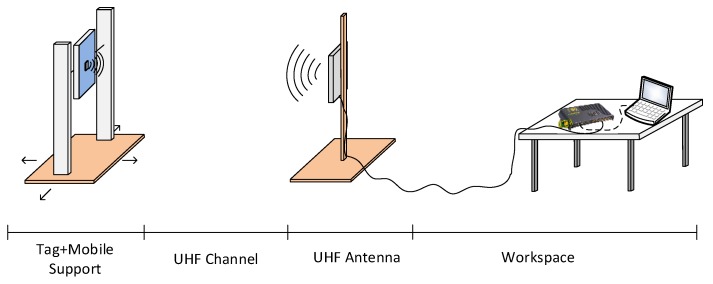
Test configuration.

In order to compare the performance of both technologies, the test configuration shown in Figure 5 was implemented for passive and active cases.

The test setup consists of a mobile support, where the RFID tag was installed over a metallic plate (45 × 15 cm^2^), and the RF antenna was connected to the RFID reader, which is controlled by a computer in order to visualize the results.

##### Passive Case

(a) Maximum coverage range

From the previous experiments presented in [32], we can observe that the range in which the power received at the tag was above the theoretical sensitivity (i.e., −10 dBm) is about 7 m, for the case of placing the tag and the antenna faced and using a vertical polarization of the antenna, achieving, occasionally, a maximum distance up to 9.5 m.

(b) Multiple readings capabilities

For multiple simultaneous readings, we can observe that only one tag of five placed at the same metallic plate was detected for a distance higher than 4 m, as shown in Table 3 [32].

(c) Performance in a not faced scenario

**Figure 6:**
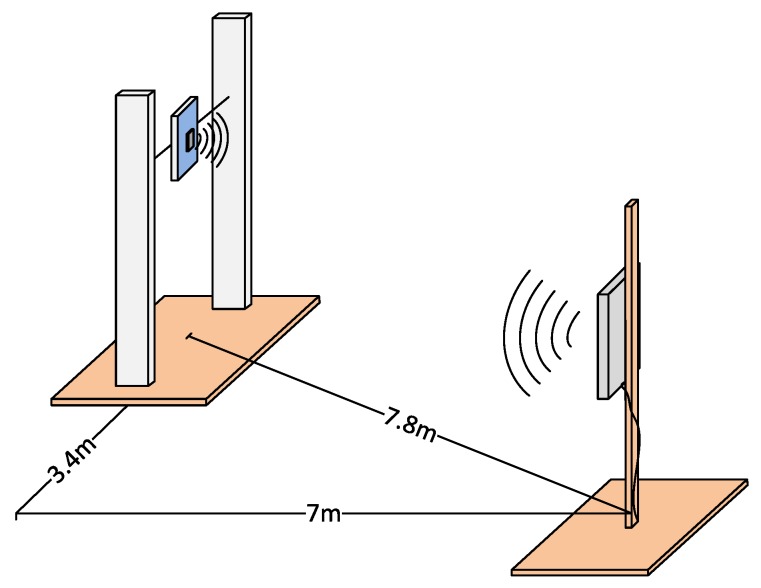
Maximum distance range for a non-faced passive tag.

Finally, starting from a position with the antenna and the tag initially faced and separated 7 m, the tag was displaced orthogonally to the line of sight (Figure 6), with the objective of observing the performance when it is not aligned with the reader. Under these conditions, the tag was detected for a maximum horizontal distance of 3.4 m.

##### Active Case

(a) Maximum coverage range

Previously to measure the coverage range for this technology, a preliminary test was conducted with the objective to evaluate the performance of the three active tags referenced in Section 4.1.1 (b) in a metallic environment, in order to select one of these models for the rest of the experiment and be compared with the passive case. All these active tags were detected when faced to the reader and separated by a distance of 7 m, which was the experimental range for passive case. However, the main observed difference between the three active tags is their behavior when placed on a metallic plate: the speed of reading of the NT02 model was enormously decreased on a metallic environment. Thereby, placing samples of NT02, RT02, and ZT02 at the same metallic plate, we only made two readings per scan for the NT02 tag, while we had obtained 68 readings without the plate. However, for the rugged models (RT02 and ZT02), the number of readings is much less decreased, mainly for the case of ZT02. These results are shown in Table 4.

The next task for the active case was to measure the maximum distance range using the rugged active tags (RT02 and ZT02) over the metallic plate. The results are shown in Table 5, for a fixed tag-floor distance of 1.5 m, being possible to observe how this maximum range is pretty longer than that in the passive case, as expected.

(b) Multiple readings capabilities

Also from the results illustrated in Table 4, it is possible to observe how the collision problems are less accused than in the passive case (see Table 3). This effect is predictable, because manufacturers of active tags usually provide anti-collision protocols (>100 tags can be simultaneously read for the proposed tags). However, this should be not a decisive factor to select the technology since usually only one tag will be placed at each traffic sign or urban facility. In any case, even if a multiple reading is required, Table 3 shows that it is also possible to read up to three and four tags placed at the same metallic plate for a distance from the reader of 4 and 2 m, respectively, using passive technology. Moreover, as will be validated by simulation in Section 4.2, the same tag is usually read several times while the vehicle is inside its cover area, thus decreasing the impact of possible collisions over the inventory process, as will be corroborated in experimental results presented in Section 4.3, where multiple simultaneous readings are performed with the reader mounted in the vehicle.

Finally, in the best observed case (i.e., using a ZT02 tag and the antenna faced at the same height and at the maximum distance range of 30 m) the number of readings starts to decrease (<3 readings/s) when the horizontal distance between the antenna and the tag is higher than 0.8 m (using the setup of Figure 6), decreasing the efficiency of the active system in comparison to the case when the transmitter and the receiver are faced (>10 readings/s). Recall that, for the passive case, tags were detected at horizontal distances up to 3.4 m. Therefore, taking into account that the reader installed in the car will be usually separated from the sidewalk a horizontal distance >2 m, it is possible to predict how the capabilities of the employed active RFID equipment will be not totally exploited for this application.

#### 4.1.3. Comparative of Results: Selection of the RFID Technology

Although active RFID technologies have some important advantages, mainly a higher sensitivity which allows a longer distance range, they also have important drawbacks, such as:

- cost per tag
- need for battery replacement and higher maintenance costs
- higher probability of interference with other ISM networks
- proprietary communications protocols

By contrast, using passive technology:

- The obtained distance range (around 7 m from static experiments) seems satisfactory for inventory of traffic signs and other urban facilities, since the identifiers will be read progressively while the reader is approaching to the tag, employing a usual vehicle speed (50–100 Km/h, i.e., 14–28 m/s) and considering the theoretical data rate (40 Kbps according to the EPC Global 1G2 ISO 18000-6C [42], i.e., around 400 tags/s for the employed tag).
- A complex anti-collision protocol is not necessary, since it is possible to detect several tags placed at the same metallic plate.

Therefore, at this point, we can conclude that active technology will probably oversize the system, exceeding the reasonable application requirements with an additional higher cost, which yields passive technologies as a more convenient option for this application, since they are open-standard systems and more cost-efficient. These conclusions will be validated by the in motion test at simulation (Section 4.2) and experimental level (Section 4.3).

### 4.2. Simulated In-Motion Results

In this section, the feasibility of the proposed link is theoretically evaluated using the radio propagation model described in Section 3. The methodology is as follows: firstly, as a function of the location of the vehicle, we calculate the direct and reflected fields at the RFID tag using Equations (6)–(8). Secondly, using (14) we calculate the probability *P_downlink_* that the power received at the tag is higher than the required threshold power. Thirdly, the calculations are repeated for the uplink (tag to reader) communication and the coverage probability *P_uplink_* is obtained. Finally, assuming that both the downlink and the uplink are independent events, the probability that the overall communication will be correct can be obtained by the product *P_coverage_ = P_downlink_P_uplink_*. In all the experiments, we consider vehicle as a point traveling at constant velocity v along a straight line. The height of the vehicle antenna from the ground is assumed to be equal to 1.3 m and the effective radiated power transmitted by the reader is set to 2 W, according to ETSI regulation EN 302–208. The sensitivity of the reader and the RFID tags are set to −80 dBm (10^−8^ mW) and −10 dBm (0.1 mW) respectively, which are realistic values for passive RFID technologies [43].

**Figure 7:**
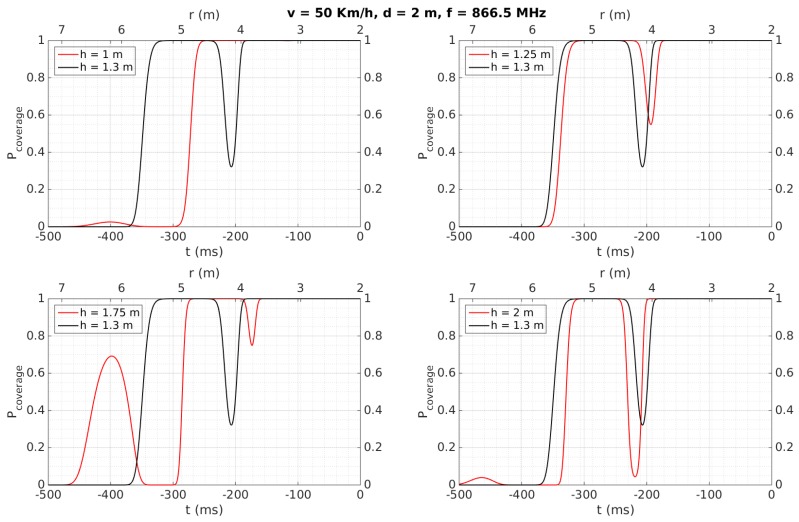
Simulation results: *v* = 50 Km/h, *d* = 2 m, *f* = 866.5 MHz.

**Figure 8:**
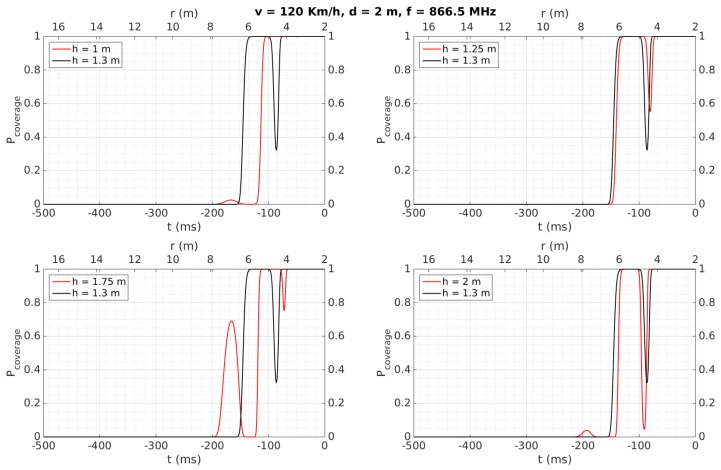
Simulation results: *v* = 120 Km/h, *d* = 2 m, *f* = 866.5 MHz.

**Figure 9:**
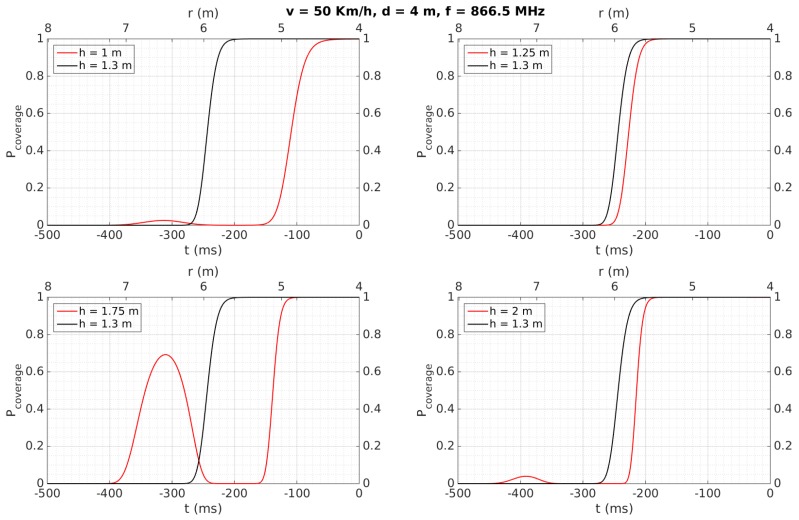
Simulation results: *v* = 50 Km/h, *d* = 4 m, *f* = 866.5 MHz.

**Figure 10:**
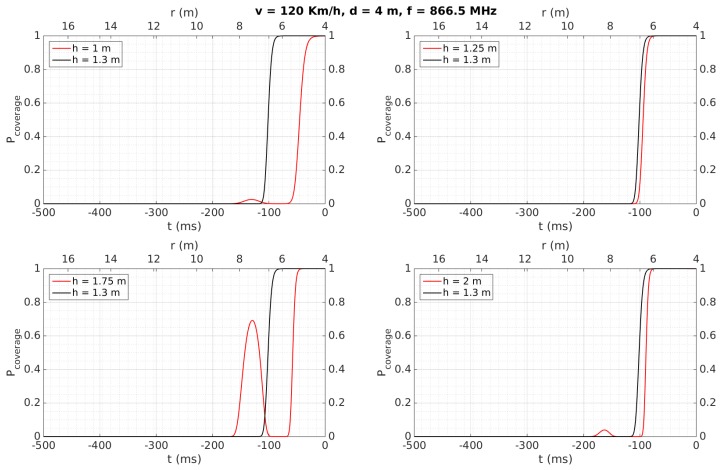
Simulation results: *v* = 120 Km/h, *d* = 4 m, *f* = 866.5 MHz.

**Figure 11:**
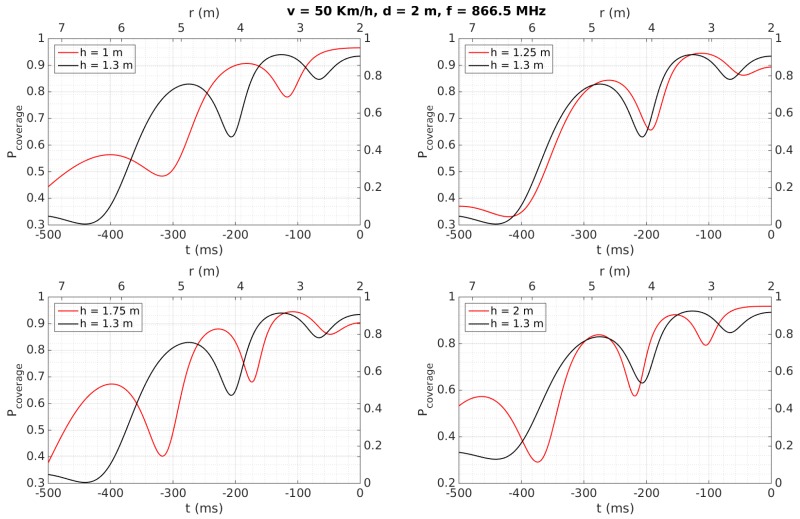
Simulation results: *v* = 50 Km/h, *d* = 2 m, *f* = 866.5 MHz in a severe multi-path environment.

**Figure 12:**
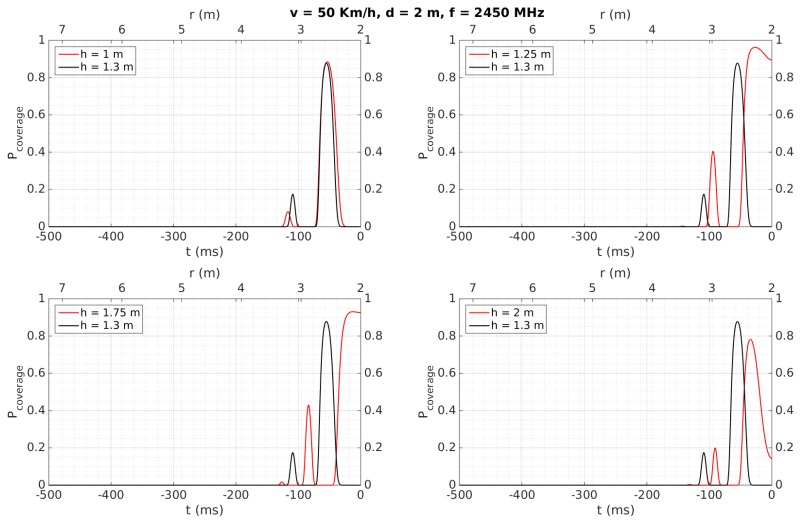
Simulation results: *v* = 50 Km/h, *d* = 2 m, *f* = 2450 MHz.

Figure 7, Figure 8, Figure 9, Figure 10, Figure 11 and Figure 12 show in red line *P_coverage_* for different design parameters and RFID tags located at several heights of the traffic sign (1, 1.25, 1.75, and 2 m for each subfigure) from the ground. For comparison, this probability is also shown in black line for the case in which the tag and the vehicle antenna are at the same height (1.3 m).

In Figure 7, the distance from the traffic sign to the road is assumed to be *d* = 2 m, the speed of the vehicle is set to 50 Km/h and the frequency of the wave equals 866.5 MHz. Furthermore, we suppose that there is no significant multipath interference, apart from the wave reflected at the road (thus we set *σ* = 0.1 *r_rms_*), and assume a vertical polarization, which optimizes the cover area, as described in [32]. Note that Figure 7 has two x-axes: ‘time taken to reach the traffic sign’ (starting at *t_0_* = −0.5 s and finishing at *t* = 0) at the bottom, and ‘linear distance between the vehicle and the sign’ (with *r = d* when *t* = 0) at the top. These axes will be used in Figure 8, Figure 9, Figure 10, Figure 11 and Figure 12 as well. Figure 7 shows, for example, that *P_coverage_* starts to be close to one for about 350 ms in the case of the tag and the antenna are faced (tag height at 1.3 m), and for about 250 ms in the case of the tag is at 1 m from the ground. Results for *h* = 1.25 m are very similar to *h* = 1.3, whereas *h* = 1.75 m presents more accused notches or blind zones due to the multi-path effect. This effect has less influence at higher heights (*h* = 2 m), where the results are more similar to the ideal case (tag and antenna faced). In general, regardless of height, there is at least 250 ms with maximum *P_coverage_* before reaching the traffic sign. Since the usually employed RFID protocol EPC Global 1G2 ISO 18000-6C provides a data rate of 40 Kbps (i.e., 400 tags/s, so it requires about 25 ms for identifying a tag), we can ensure that the complete radio link will be reliable for the whole transaction time, and that the tag can be read about 10 times during the passage of the vehicle.

Therefore, even considering a high density of tags (such as the case of an urban environment, where the facilities to be inventoried are often close to each other), this obtained performance allows to read several tags at the same cover area (around 5 m in the simulations). Additionally, due to the possible presence of several tags close to the reader, the time required for managing possible collisions has to be considered. This time leads to an additional reading time (25 ms) for each given collision [44], i.e., basically the reading process has to be repeated. Therefore, the obtained total cover time (about 250 ms, see Figure 7) may be still enough to correctly read each tag even in the case of several collisions.

Similar curves are presented in Figure 8, illustrating the case when the vehicle increases its speed up to 120 Km/h. Compared to Figure 7, it is evident that the curves are time-compressed, meaning that the period during which the link is reliable may decrease to around 100 ms. The analysis in function on the different heights is similar to the case of Figure 7. Also, from a similar analysis as described for the previous case, this time of 100 ms would allow tag identification as well. 

Cases illustrated in Figure 9 and Figure 10 are very similar to those shown in Figure 7 and Figure 8, respectively. The difference consists in an increase in the distance between the tag and the antenna to *d* = 4 m, which leads to reducing the time with a *P_coverage_* ≈ 1 to 200 ms (at 50 Km/h) and <100 ms (at 120 Km/h).

Figure 11 shows the results for a severe multi-path scenario (the parameter σ is set to *σ* = *r_rms_*), setting a speed of 50 Km/h, as an approximation of an urban environment with a high density of reflections. Even for this case, *P_coverage_* reaches a value close enough to one during at least 100 ms for the different studied heights, so that a correct detection is feasible even for this very harsh scenario.

Finally, Figure 12 shows the simulation results by using a carrier frequency of 2450 MHz and no significant multipath interference, apart from the ray reflected on the road. We observe that *P_coverage_* is maximum for a short time interval, in which the antenna is very close to the tag, its duration being lower than the time of 25 ms required by the protocol. Therefore, the 866.5 MHz band is a better option by using passive technologies, whereas the 2.45 GHz band would only result feasible when active technologies are employed, whose transmission power is much higher than the passive case. Thereby, these results support the selection of the passive RFID band proposed in Section 4.1.

### 4.3. Experimental In-Motion Validation

**Figure 13:**
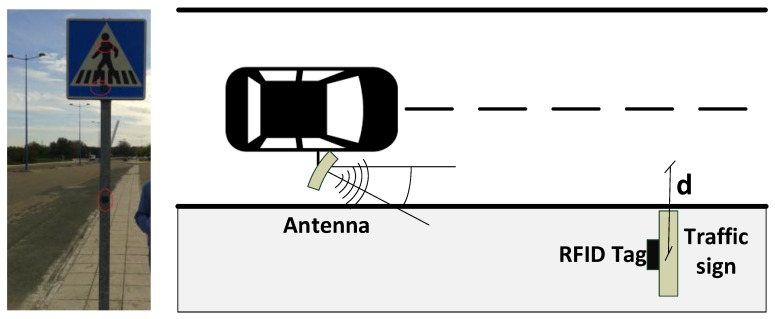
Tags over the traffic sign (**left**) and configuration setup (**right**).

**Figure 14:**
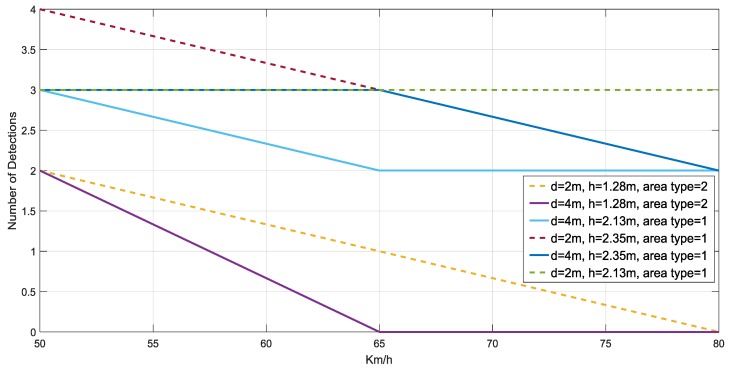
Number of detections for each studied case.

In this real scenario, three passive tags are attached over a real traffic sign and its metallic pole at three different heights (1.28, 2.13, and 2.35 m, as shown in Figure 13 (left)). The reader is installed in the car, with the antenna at about 1.5 m above the floor and pointed to the traffic signs from the open window of the vehicle. In the configuration illustrated in Figure 13 (right), the distance *d* between the traffic sign and the antenna varies from 2 to 4 m in the experiments. Some tests were performed at different speeds, whose results are showed in Table 6 and Figure 14. It is possible to observe that the results are satisfactory at any usual speed of the car without interfering in the traffic flow, as predicted by the simulations results. Moreover, from Figure 14, we can observe a general tendency to decrease the number of detections when the speed and the distance *d* are increased, where the field “area type” is *1*, when the tag is placed over the metallic traffic signal, or *2*, when the tag is over the metallic pole.

Table 6 also shows that tags were always detected when the vehicle speed was 50 Km/h, considering a reliable detection when the tag is read at least two times. They are detected for any vehicle speed as well when they are placed over the traffic sign (2.13 and 2.35 m), instead of being placed over the metallic pole (1.28 m), even for a distance *d* = 4, which implies the system would work properly even in the case of having to maintain a certain separation from the traffic sign, which may occur in a multi-lane road scenario. This better performance at 2.13 and 2.35 m can be mainly attributed to the fact that, at higher heights, multi-path effects have less influence and the performance is more similar to the case where the antenna and the tag are faced, as was observed in Section 4.2 for the simulation results for *h* = 2 m. Moreover, the performance is worse when the tags’ heights are lower than the antenna’s height, which was also observed in the simulation results illustrated in Figure 7, Figure 8, Figure 9, Figure 10, Figure 11 and Figure 12 for *h* = 1 m, where a higher time is required to reach *P_coverage_* = 1, with this being more critical drawback at higher speeds.

Besides the effect of placing the tags at higher heights, another reason to explain this better performance at 2.13 and 2.35 m is that these tags are placed over the traffic sign, whose area is wider than that of the metallic pole, thus increasing the original ground plane of the RFID tag and improving the performance of its antenna [45].

Note that tags may be also identified attached to urban facilities with small back-placed metal plates, since the performance is satisfactory in the most similar cases in Table 6, i.e., when the speed is limited at its maximum for these urban environments (50 Km/h) and the tags are attached to the smallest metallic surface (height of 1.28 m). Also, the obtained results can be extended to the inventory of other urban facilities with several tags placed very close, since all of them are detected in any case at 50 Km/h, as predicted in Section 4.2, leading to a reliable system for the applications where nearby items have to be detected without collision problems.

## 5. Discussion: Comparison with Related Works

A comparison with the similar works [9,10,11,12,24] referenced in Section 1 is detailed in Table 7. To the best of our knowledge, these approaches are the most related to that proposed in this paper in terms of their applications. Having said that, we observe that some of their features (e.g., the frequency band or the selected technology, active RFID in most cases) are clearly distinct from those proposed in the present paper. Therefore, Table 7 is focused on highlighting how employing the selected technology, band, and equipment is possible to meet the requirements for the intended inventory-making application, with some benefits (such as costs) over the similar previously presented approaches. First of all, note that we present a system dedicated to inventory and location purposes, whereas that [9,10,24] propose ADAS implementations to display digital sign traffics, and [11,12] proposes the attachment of RFID tags at traffic signs as part of a high accuracy vehicle speed controller system.

Apart from the present work, only [9] proposes the use of passive RFID technologies. However, ref [9] is implemented by using tags with a short distance range, no ruggedized and no designed for metallic environment applications. This implies that these tags are placed at the road lane instead of on the traffic sign, unlike the others referenced works, losing the capability of detecting displacements or thefts of the traffic signs. Moreover, the tags used in [9] limit the speed of the car to 20 Km/h, which would interfere with the normal traffic flow in many cases. Finally, although the main advantage of these tags is their price, this cost difference is decreased considering that [9] employs three tags per traffic sign, whereas the others works employ one. In fact, [24] presents an improvement of the encoding proposed in [9] in order to reduce the number of tags.

Regarding the active RFID systems [10,11,12], it is possible to observe that the distance range achieved by the present work is lower, as expected, but still enough to detect all the traffic signs and urban facilities when using a vehicle running at a normal speed, while benefiting, at the same time, from the advantages of passive RFID technologies, such as costs, tag size, and no need for battery replacement. An additional feature of [12] is the use of two independent readers for redundancy, in order to avoid the occasional missed tags detections that may occur by using only one reader. This feature can be also implemented in our system, since the employed reader is equipped with four antenna ports that can be orientated in multiple directions. This possible future direction could be useful with the objective to detect tags placed at the back of the traffic signs left behind by the vehicle, or at the front of the traffic signs installed in the oncoming circulation roads. Moreover, this extension would help avoid a possible limitation of the proposed system regarding occasional occlusions of the line of sight by metallic elements, which could happen, for example, when a truck is stopped in front of the traffic sign. Other future direction related with the use of several antennas is to validate the system for traffic signs placed over the highways, which is a scenario that has not been experimentally tested yet. However, note that the tags in this scenario would be placed at higher heights, so the effects of multi-path propagation are expected to be not significant. Moreover, in this case RFID tags will be placed over larger metallic plates, improving the performance of its antenna. Therefore, pointing the antenna properly, a correct detection of these traffic signs is expected, since it is, a priori, a favorable scenario. Therefore, this implementation based on multiple antennas could lead to a more effective inventory process and, in general, improve the efficiency of any application such as the use of the system for navigation purposes.

Moreover, the proposed tag presents the most compact size and the higher ID capacity for most of cases, which facilitates the implementation of the inventory and location functionalities, and its extension, as a future direction, to other applications or migrations as well, for example, adapting this ID to the inventory of other urban facilities. In fact, by using the proposed work as a basis, it would be possible to implement the functionalities of [9,10,11,12,24] for smart city and advanced cruise applications, taking advantage of passive RFID technologies. In addition, note that, by using an open wireless communication standard, the features of the reader could be improved in the future with the advance of the technology, since the installed tags would still work with new technologies.

In summary, from the discussion of the data showed in Table 7, we can conclude that the feasibility of the system has been demonstrated for the intended purposes, fulfilling the requirements for the application, since the longer coverage range provided by active RFID is not necessary, so it is possible to avoid its main drawbacks regarding costs, maintenance tasks, and use of proprietary protocols. Additionally, in comparison with the passive case [9], a better performance has been obtained mainly because of the features of the selected equipment.

Finally, a similar study is detailed in Table 8, with the objective to present a comparison with other passive RFID systems working at the same frequency band and using similar versions of the wireless communication standard employed in this work, but with different road/vehicle applications [19,20,21,22,23]. Note that several features have been omitted for Table 8 (regarding Table 7) for different reasons:

- Tag ID capacity: this value is not provided by these papers. Only [20,23] use the maximum value given by the standard (512 bits) for the presented calculations, models, and simulations.
- Price/tag: this value is not provided, but we can assume similar prices in the case that these works employ ruggedized passive tags.
- Maximum reading and data rate: only [19] gives a value of 100 tag/s and [20] values of 62.5 tags/s and 32 Kbps. Since these values depend on the tag ID capacity and the employed standard, they have been omitted in Table 8 as well.
- Tag area: this value only is given by [22] (98 × 12 mm^2^). Since all these works use the same technology, also we can assume similar areas.

Regarding distance range, we can observe how the proposed work achieves a satisfactory performance comparing with the referenced works, mainly because they are focused on placing multiple tags on the floor to locate accurately the vehicle passing over them [19,22], or to identify vehicles placing the reader just on the floor to identify the tag attached to the vehicle passing over or very close to the antenna [20,21,23]. In other words, they do not have the purpose of identifying a tag relatively far from the car. Only [21], which performs an analysis at simulation level, achieves up to 10 m, with the vehicle running up to 90 Km/h. Also from theoretical calculations, [23] achieves a distance range of 6 m, which is similar to that obtained in the present work. Regarding the speed of the car, it is possible to observe how [22] achieves a very low value because this work is focused on minimizing the positioning error (<1.35 cm) of a mobile vehicle in an indoor environment, whereas [19,20,21,23] achieve higher speeds at theoretical and simulation levels, as described in Table 8. Note that the present work is able to detect the tags also running at 120 Km/h (see Figure 8 and Figure 10) at simulation level, in the same order of some of these approaches. Therefore, comparing with the previously presented works using the same technology and frequency band, we can conclude that the proposed system presents, as main novelties, its use for inventory of traffic sings applications and the experimentally achieved values of distance range and speed of the car.

## 6. Conclusions

In this paper, we have proposed a design approach for a wireless cost-effective system based on passive RFID technologies with real-time inventory and location purposes. The proposed system targets applications such as the identification of traffic signs or urban facilities in scenarios where the visibility is reduced and, therefore, image processing based systems are not reliable. The proposed system implements a self-contained and self-updated data base, providing high scalability and low infrastructure maintenance outlays, reducing the timing and costs of the whole inventory process, and without interfering with the normal traffic flow. Additionally, exploiting the implemented inventory infrastructure, the EPC tags can be also employed to implement a low-cost and open source location and navigation system, by mapping these identifiers to georeferenced coordinates in order to present the exact location in a map by using a web interface. Since the performance of passive RFID is not evident for the proposed in-motion application at a vehicle’s speed, due to its lower coverage range, a theoretical model of the wireless communication link and tests at simulation and experimental levels have been performed in order to estimate this coverage range, the speed limits, and the emplacement of the tags at the traffic signs. These contributions have allowed to validate the selection of the RFID technology, describing the convenience of using passive tags over active tags for this application. Finally, we have presented the in-motion experimental results of the on-board system, demonstrating that the proposed approach based on passive RFID is an appealing low-cost and low-complexity alternative for inventory and location purposes on road and urban scenarios, finding application within the framework of the new generation driving-aid and infrastructure-to-vehicle systems.

## Figures and Tables

**Table 1:** RFID systems for road applications.

Work	Application	Technology	Frequency (MHz)
[9] Sato, 2006	In-vehicle traffic sign detection	Passive	13.56
[10] Mariut, 2012	In-vehicle traffic sign detection	Active	2400–2483
[11] Paul 2012	Traffic sign alert system	Active	867
[12] Pérez, 2010	Intelligent speed controller	Active	433
[13] Song, 2014	Vehicle positioning in tunnels	Active	417.05–435.9
[14] Wang, 2014	Vehicle positioning	Active	902–928
[15] Prinsloo, 2016	Vehicle location for IoT applications	Passive	0.125–0.134
[16] Ali, 2017	Traffic light recognition	Passive	13.56
[17] Mandal, 2011	Road traffic congestion monitoring	Active	2400
[18] Sundar, 2015	Traffic congestion control, ambulance Clearance and stolen vehicle detection	Passive	0.125
[19] Digiampaolo, 2012	Autonomous vehicle location	Passive	870
[20] Hoffman, 2015	Vehicle identification	Passive	920–923
[21] Larionov, 2017	Automatic vehicle identification	Passive	860–960
[22] Park, 2013	Recognition of vehicle position	Passive	917–923.5
[23] Wang, 2017	Vehicle identification	Passive	910–920

**Table 2:** Codification of the EPC frame.

Field (Used Name)	Length (bits)	Description
Signal (Sg)	10	Indicates the type of signal.
Signal number (SN)	10 ^1^	Identifier of the traffic sign within its stretch.
Stretch (St)	8 ^2^	Identifies the stretch within a road.
Road (Rd)	26 ^3^	Road identifier.
Kilometer (Km)	11 ^4^	Indicates the kilometric location on the road of the traffic sign.
Meter (M)	10	Indicates the distance in meters from the start of the kilometer to the traffic sign.
Installation date (InD)	10 ^5^	Indicates the date when the traffic sign was installed, in order to know if it has to be replaced.
Cyclic redundancy check (CRC)	5	Code to detect possible errors in the reception of the EPC.

^1^ This length will be oversized, since it allows to code around five traffic signs per meter. ^2^ This length is 8 bits because the longest road in Spain (named N-340) is 1248 Km long, so it is composed of 250 stretches. ^3^ This length is 26 bits because the usual format name for a Spanish road is composed of two letters and a three-digit number, requiring 16 bits for the letters (using an ASCII 8-bit code) and 10 bits for the number. ^4^ This length is 11 bits because the maximum length is 1248 Km. ^5^ This length is 10 bits in order to use 4 bits to codify the month and 6 bits to codify the year.

**Table 3:** Multiple readings for the passive case.

Distance (m)	Detected Tags
0.1	1, 2, 3, 4, 5
1	2, 3, 4, 5
2	2, 3, 4, 5
4	3, 4, 5
6.7	5

**Table 4:** Readings for the active tags in a 3-s test time.

	Readings without Metallic Plate	Readings with Metallic Plate
NT02	68	2
RT02	37	26
ZT02	37	30

**Table 5:** Maximum distance range for active tags.

Reader Antenna Distance from Floor (m)	Maximum Range for RT02 (m)	Maximum Range for ZT02 (m)
~0	25	30
0.75	28	31
1.5	30	34

**Table 6:** In-motion results.

Speed (Km/h)	Distance d (m)	Height (m)	Detection	Number of Detections
50	2	1.28	Yes	2
50	4	1.28	Yes	2
50	2	2.13	Yes	3
50	4	2.13	Yes	3
50	2	2.35	Yes	4
50	4	2.35	Yes	3
65	2	1.28	No	1
65	4	1.28	No	0
65	2	2.13	Yes	3
65	4	2.13	Yes	2
65	2	2.35	Yes	3
65	4	2.35	Yes	3
80	2	1.28	No	0
80	4	1.28	No	0
80	2	2.13	Yes	3
80	4	2.13	Yes	2
80	2	2.35	Yes	2
80	4	2.35	Yes	2

**Table 7:** Comparison between traffic signs detection systems based on RFID.

	[9]	[10]	[11]	[12]	This Work
**Type**	Passive	Active	Active	Active	Passive
Application	In-vehicle traffic signing	In-vehicle traffic signing	Traffic sign alert system	Intelligent speed controller	Inventory and location
Carrier frequency (MHz)	13.56	2400–2483	867	433	866.5
On average read range (meters)	0.4	30	10	23	7 ^1^
Speed of the car (Km/h)	<20	<100	<90	<24	<80 ^2^
Tag ID capacity (bits)	64	24	192	-	96
Price/tag (US$)	1.31	-	20	10–20	3.3
Maximum reading rate (Tags/s)	50 ^3^	10 ^3^	-	-	400 ^3^
Data rate (Kbps)	3.2	250	-	-	40
Tag area (mm × mm)	76 × 45	-	80 × 40	123 × 80	51.5 × 47.5

^1^ Experimental maximum distance range from the static experiments reported in Section 4.1.2. For simulations, the distance range with *P_coverage_* = 1 is 5 m approximately. ^2^ For the case of attaching the tag over the traffic sign. When the tag is placed over the pole of the traffic sign, at a lower height, the speed is limited to 50 Km/h. ^3^ These values are the theoretical maximum rates given by the specifications.

**Table 8.** Comparison between passive UHF RFID based systems

	[19]	[20]	[21]	[22]	[23]	This Work
**Application**	Autonomous vehicle location	Vehicle ID	Automatic vehicle ID	Recognition of vehicle position	Vehicle ID	Inventory and location
Carrier frequency (MHz)	870	923	860–960	902.75–927.25	900–920	866.5
On average read range (meters)	0.5	4	10 ^1^	0.8	0.5 ^2^	7 ^3^
Speed of the car (Km/h)	<128 ^1^	<300 ^4^	<90 ^1^	<1.8	<100 ^4^	<80 ^5^

^1^ Value given by simulations. ^2^ Distance between the antenna on the floor and tag at the car. The theoretically calculated maximum distance range is 6 m. ^3^ Experimental maximum distance range from the static experiments reported in Section 4.1.2. For simulations, the distance range with *P_coverage_* = 1 is 5 m approximately. ^4^ Value theoretically calculated or extrapolated. ^5^ For the case of attaching the tag over the traffic sign. When the tag is placed over the pole of the traffic sign, at a lower height, the speed is limited to 50 Km/h.
